# CD39 and CD73 as Promising Therapeutic Targets: What Could Be the Limitations?

**DOI:** 10.3389/fphar.2021.633603

**Published:** 2021-03-11

**Authors:** Ana Maria Oliveira Battastini, Fabricio Figueiró, Daniela Bitencourt Rosa Leal, Pedro Henrique Doleski, Maria Rosa Chitolina Schetinger

**Affiliations:** ^1^ Departamento de Bioquímica, Universidade Federal do Rio Grande do Sul, Porto Alegre, Brazil; ^2^ Programa de Pós-Graduação em Ciências Biológicas: Bioquímica, Universidade Federal do Rio Grande do Sul, Porto Alegre, Brazil; ^3^ Programa de Pós-Graduação em Ciências Biológicas: Bioquímica Toxicológica, Universidade Federal de Santa Maria, Santa Maria, Brazil

**Keywords:** CD39, CD73, inhibitors, purinergic system, therapeutic approach, limitations

## Introduction

The search for specific inhibitors of CD39 (ectonucleoside triphosphate diphosphohydrolase-1, NTPDase1, apyrase, ATPDase, ENTPD1, EC 3.6.1.5) and CD73 (5′-ribonucleotide phosphohydrolase, ecto-5′-NT, CD73, eNT, EC 3.5.1.5) is long underway. However, the history of this search has revealed crosstalk among components of the purinergic system, including nucleotides and nucleosides, enzymes and receptors, and the extracellular environment. This report aims to present an opinion about key features of inhibitors and antibodies against CD39/CD73 discussing their applications and limitations. Certainly, many questions have the potential to emerge, which are likely to be in accordance with new and old points of view. However, the most important questions raised in this opinion piece are: 1) what are the limitations of CD39 and CD73 inhibitor use? and 2) What information is needed to enhance our understanding of the complex purinergic network that allows cells to avoid compensatory mechanisms between cell components and the surrounding microenvironment?

### NTPDase1/CD39 Inhibitors

The first step in the extracellular metabolism of ATP involves CD39, which hydrolyses ATP and ADP to AMP (Burnstock, 2017). The search for new, potent and specific inhibitors of CD39 has been a goal of many researchers throughout recent decades. In 2006, Robson and colleagues (Robson et al., 2006) observed that the failure to discover and identify specific inhibitors of NTPDase were major impediments to making further discoveries. The first inhibitors of CD39 identified are considered classical inhibitors, and include sodium azide (NaN3), suramin, chelators (EDTA and EGTA), ARL67156 (6-N, N-diethyl-D-b,g-dibromomethylene ATP), 8-BuS-ATP (8-thiobutyladenosine 50 –triphosphate) and BG0136 (1-naphthol-3, 6-disulfonic acid). These inhibitors may increase ATP and ADP levels to enhance purinergic signaling. However, inhibitors capable of binding to P2 receptors antagonize their effects (for example, suramin) (Munkonda et al., 2007). The fact that these inhibitors are not selective for CD39 is a limitation of their use. However, classical inhibitors are very useful for characterizing CD39 activity in different cell types (Battastini et al., 1991; Leal et al., 2005).

Among the new classes of CD39 inhibitors, the most studied are polyoxymetalates (POM´s) (Müller et al., 2006). These compounds have limited cell uptake, act mainly in the extracellular environment, and have a high degree of stability at physiological pH values (Sang-Yong et al., 2015). Studies indicate that POM´s induce conformational changes in enzymes, and consequently, prevent nucleotide hydrolysis (Zebisch et al., 2012). The most potent inhibitors of CD39 are compounds called POM-1 and POM-5 (Sang-Yong et al., 2015). Although POM-1 and POM-5 are potent enzyme inhibitors, they have low selectivity for CD39, and also inhibit activities of NTPDase-2, NTPDase-3, and NPP-1. While POM-5 is the most effective CD39 inhibitor, it only moderately inhibits other NTPDase isoforms, POM-1 has similar inhibition activity against NTPDase isoforms, and most strongly inhibits NPP-1 (Müller et al., 2006; Wall et al., 2008; Bastid et al., 2015; Sang-Yong et al., 2015). Further, POM-1 was the only POM assessed *in vivo* in different tumor models (Zhang et al., 2019). The rationale for POM-1 studies includes the fact that the molecule is well tolerated and is currently the only POM sold commercially.

Recent studies have aimed to enhance production of low molecular weight inhibitors with high selectivity for CD39 including Schiff bases of tryptamine (SBT´s) and quinoline derivatives (QD´s) (Hayat et al., 2019; Kanwal et al., 2019). Among SBT´s, the SBT-C6 compound strongly inhibits CD39, but with a low degree of selectivity. The inhibitor also inhibits other isoforms of NTPDases. On the other hand, SBT-C1 selectively inhibits CD39. Thus, it is a promising agent for *in vivo* studies (Hayat et al., 2019). However, SBT’s competitively inhibit, and thus, their use for inhibiting CD39 in the most requested microenvironments (ATP-rich sites) may be limited. QDs, on the other hand, include two selective CD39 inhibitors (compounds QD-3F and QD-3T), which have high inhibition efficiencies and are also promising agents for future studies. However, the type of enzymatic inhibition induced by the QD compounds has not yet been determined. It is known that some QD compounds non-competitively inhibit CD39, and it is possible that these compounds are superior to SBT´s (Kanwal et al., 2019).

In terms of purinergic signaling and the action of ectonucleotidases, POMs are noted for their ability to act exclusively in the extracellular environment. In contrast, due to their similarities with purines, SBTs and QDs can enter cells and may induce intracellular changes. Studies assessing the toxicity of these types of new compounds are lacking. Further *in vivo* studies and more advanced clinical tests have the potential to confirm the effectiveness SD and QD inhibitors in pathological processes.

### Ecto-5′-NT (CD73) Inhibitors

The CD73 enzyme is the main source of extracellular adenosine, and it point of convergence between the canonical (through NTPDases) and non-canonical (through CD38/CD203a) pathways. In this way, CD73 connects the purinergic signaling pathway, on one side via ATP (P2 receptors) and on the other side via adenosine, which, in general, produces opposite effects by activating P1 receptors. Preliminary studies that assessed CD73 inhibitors aimed to characterize and clarify its kinetic properties. However, further studies revealed the role of the enzyme in the control of purinergic signaling in different pathological processes such as cardiovascular diseases, autoimmune processes, cancer and other diseases. These findings indicated a potential use for the enzyme as a novel therapeutic target. The first endogenous, powerful, competitive CD73 inhibitors were ATP and ADP (Burger and Lowenstein, 1970; Sullivan and Alpers, 1971). Burger and colleagues (1970) also reported that the nucleotide analogue α, β-methylene adenosine 5′-diphosphate (APCP) was a more potent inhibitor of CD73 than ADP and ATP. This ADP analogue remains one of the strongest known inhibitors of CD73. However, some of its characteristics such as its low bioavailability, low metabolic stability and off-target effects limit its therapeutic use (Ghoteimi et al., 2019). For this reason, APCP became a prototype for drug development (Bhattarai et al., 2015; Corbelini et al., 2015; Bhattarai et al., 2020). In addition, the recent availability of the crystal structures of ecto-5′-nucleotidases has led to the development of numerous docking and virtual screening studies (Knapp et al., 2012; Bhattarai et al., 2019; Viviani et al., 2020).

Available extracellular adenosine mediates immune evasion, which facilitates tumor growth and metastasis. This process has been the focus of numerous recent cancer studies (Antonioli et al., 2017). Some natural products capable of inhibiting CD73 have been reported to possess biological effects that include anticancer activities (Braganhol et al., 2007; Rockenbach et al., 2013). However, the search for natural products has become less intense than identifying synthetic products with similar activities (Dumontet et al., 2018; Iqbal et al., 2020). Recently, extensive studies of structure-activity relationships, structure-based drug design, and the optimization of pharmacokinetic properties culminated in the discovery of several different APCP analogues. To our knowledge, Prof Christa Müller’s group has found the most potent inhibitors of CD73, which are structurally related to the APCP skeleton. Bhattarai and cols (2015) prepared a series of selective and potent CD73 inhibitors with Ki values in the low nanomolar range in good yields and high purity using a multistep reactions. Importantly, the new compounds displayed high selectivity relative to other ecto-nucleotidases and ADP-activated P2Y receptors. More recently, the same group synthesized 5′- O-[(phosphonomethyl)phosphonic acid] derivatives. These uridine- and cytosine-derived α,β-methylene diphosphonates represent an entirely new class of CD73 inhibitors that proved to be potent inhibitors of rat and human CD73 with Ki values in the low nanomolar range, too (Junker et al., 2019). In addition, an orally bioavailable small-molecule CD73 inhibitor (OP-5244) was able to reverse immunosuppression via the blockage of adenosine production (Du et al., 2020). Recent published data has revealed another highly potent (Ki = 5 pM) and selective inhibitor of ecto-5′-NT/CD73 (AB680). Importantly, AB680 is well tolerated and exhibits a pharmacokinetic profile suitable for intravenous administration in humans (Bowman et al., 2019; Lawson et al., 2020). This and another small molecule inhibitor (LY3475070) are currently being evaluated in phase one clinical trials. In addition to APCP based inhibitors of CD73, descriptions of numerous other inhibitors of the enzyme can be found in the literature (Figueiró et al., 2014; Baqi, 2015; Yang et al., 2017; Ghoteimi et al., 2019; Iqbal et al., 2020; Viviani et al., 2020). Finally, nucleoside analogues with two carboxylate groups and benzothiazine derivatives are CD73 inhibitors for treating cancer that have been patented (Gong et al., 2018; Ghoteimi et al., 2019).

In summary, the field of research on CD73 inhibitors has been quite intense and, in our opinion, the use of small molecules to inhibit CD73 activity for clinical use is promising. It is likely that these therapeutics will be used in association with established chemotherapies and/or new immunotherapies strategies.

### CD39/CD73 Axis as Pharmacological Target for Immunotherapy

No commercially available antibody blocks CD39. However, some antibodies with this activity are in preclinical or clinical trials in several cancer models. In preclinical trials, the CD39-blocking antibody OREG-103/BY40 alone, with the A2AR antagonist (SCH58261), or with ARL67156 was shown to improve CD4 and CD8 T-cell proliferation and enhance cytotoxicity induced by CD8 T-cells and NK cells against tumoral cell lines (Bastid et al., 2015), demonstrating its potential antitumor effects. These effects have been associated with reduced adenosine production, which occurs as a consequence of CD39 inhibition. The OREG-103/BY40 monoclonal antibody (mAb) was capable of blocking extracellular enzymatic activities and has been tested clinically in relation to its efficacy (Nikolova et al., 2011; Perrot et al., 2019). The anticancer potential of another antagonistic mAb (clone 9-8B) was assessed, which revealed that the compound slowed tumor development in a sarcoma model (Hayes et al., 2015).

A recent clinical study in phase one assessed IPH5201, an anti-CD39 antibody. The compound has been administrated as monotherapy, or in combination with immune checkpoint inhibitors, to enhance the antitumor immune response. Unlike BY40, IPH5201 blocks extracellular and soluble CD39, reducing ATP hydrolysis and adenosine production. This antibody increased antitumor activity when administrated with chemotherapeutic drugs, such as oxiplatin (Perrot et al., 2019). Other antibodies including anti-CD39, TTX-030, and SRF617 have been tested in phase one clinical trials, both as single agents and in combination with chemotherapy, with the aim of improving the antitumoral response (Moesta et al., 2020).

Since it was observed that CD39 deficient mice have altered platelet function, thrombotic events could be a concern because blocking CD39 may affect other biological functions of purinergic signaling. Activated platelets also release factors that contribute to the degradation of the extracellular matrix and the preparation of the metastatic niche (Palacios-Acedo et al., 2019), that can be a risk of CD39 blockade. However, it was recently shown that anti-CD39 administration in mice did not promote thrombosis (Allard et al., 2020). CD73 stimulate VEGF release in tumor cells through adenosine, inducing the angiogenesis in the tumor microenvironment, which facilitate the metastasis (Allard et al., 2014). Several studies have demonstrated the correlation between CD73 expression and/or activity with tumor cells capacity of adhere to extracellular matrix and invade different tissues. Then, CD39 and CD73 blockade can beneficially reduce tumor growth and metastasis by reducing tumor angiogenesis (Antonioli et al., 2021).

Moreover, it has been extensively shown that inhibiting CD73 may directly improve outcomes of conventional therapies by directly targeting cancer cells or by decreasing adenosine levels and, consequently, indirectly promoting antitumor-effector immune cells in the alleviation of the immunosuppressed microenvironment (Azambuja et al., 2019). In addition to clinical trials involving synthetic inhibitors, (e.g. LY3475070 and AB-680), mAbs are also currently being evaluated for activity against CD73, (e.g. oleclumab, CPI-006, AK119, TJ004309, NZV930, BMS-986179). Alternatively, bifunctional mAb (GS-1423) that target CD73 and TGF-β pathways have been assessed. These molecules are being investigated for use mostly as combined therapies for advanced cancer patients (www.clinicaltrials.gov). Monoclonal antibodies have been displayed fewer off target effects than natural or synthetic CD73 inhibitors. In this sense, in comparison to mAbs, classical molecular inhibitors AMPCP (CD73) and ARL67156/POM-1 (CD39) and their derivatives show inhibition capability over other purinergic targets and have been mostly studied in the preclinical field. In addition to specificity, pharmacokinetic characteristics and unexplored side-effects have contributed to the low translationality of small synthetic inhibitors (Jeffrey et al., 2020).

It has been widely demonstrated that CD39 and CD73 may be overexpressed in cancer cells, different immune cell subsets and stroma cells working in a coordinated way to arrest antitumor immunity via increasing levels of adenosine in the tumor microenvironment (TME) (Allard et al., 2017). Thus, especially from two pioneering works (Stagg et al., 2010; Sun et al., 2010), targeting the CD39/CD73 axis has become an immunotherapeutic strategy that aims to reduce adenosine levels and allow proinflammatory ATP to shift the cells from a protumoral to antitumoral immune response (Perrot et al., 2019). Considering the potential role of CD73, and possibly CD39, as adhesion molecules, the efficiency of blocking these proteins by specific antibodies may not be related only to their enzymatic activities and adenosine production. For example, some studies have shown that the blockage of CD73 may prevent its adhesion to extracellular matrix affecting the potential migration of cancer cells, an important event of tumor invasiveness (Zhou et al., 2007; Sadej et al., 2008; Cappellari et al., 2012). Although adverse effects must be carefully evaluated, the strategy considers that the enzymes are overexpressed in the TME and, therefore, drugs’ benefits can outweigh the possible risks. Cutting-edge research such as microRNA (Zhou et al., 2019), siRNA (Azambuja et al., 2020) interventions, antibody-drug conjugates (Abdollahpour-Alitappeh et al., 2019), mAbs or microRNA delivery by active targeting nanotechnology (Lin et al., 2020) may be the next wave of purinergic interventions used in the treatment of cancer.

## Concluding Remarks

The purinergic system is an ancestral signaling system. Therefore, it can be expected that the system developed the capacity to participate in crosstalk with another signaling systems and components of the cell to create compensatory mechanisms to ensure cell survival. In fact, this ancestral system is able to regulate signaling processes by enhancing or decreasing enzyme activities and expression levels, nucleotide and nucleoside levels, and binding and expression levels of specific receptors. Then when using inhibitors, some features must be considered. First, the selectivity of the inhibitors is important. It must be ensured that inhibitors alter only the target component of the purinergic system. For example, an inhibitor should target one specific enzyme, but not block receptors of other cytosolic enzymes. Second, unexpected responses may occur as a consequence of crosstalk with the surrounding environment. The highly intricate TME and CD73/CD39 axis allows for the modulation of immune and cancer cells.

In our opinion, the use of inhibitors or immunotherapy for regulating CD39/CD73 axis could be a promising therapeutical approach by promoting the increase in the ATP and the decrease in the adenosine levels, causing strong immune and anti-tumor responses. In addition, inhibiting the adenosinergic system may modulate this immune checkpoint leading to a synergistic effect with the conventional anticancer treatments such as chemo- and radiotherapy and even with current immunotherapies.
